# Slit2-Mediated Metabolic Reprogramming in Bone Marrow-Derived Macrophages Enhances Antitumor Immunity

**DOI:** 10.3389/fimmu.2021.753477

**Published:** 2021-10-28

**Authors:** Kirti Kaul, Martin Benej, Sanjay Mishra, Dinesh K. Ahirwar, Marshleen Yadav, Kristin I. Stanford, Naduparambil K. Jacob, Nicholas C. Denko, Ramesh K. Ganju

**Affiliations:** ^1^ Comprehensive Cancer Center, The Ohio State University, Columbus, OH, United States; ^2^ Department of Pathology, The Ohio State University, Columbus, OH, United States; ^3^ Department of Radiation Oncology, The Ohio State University, Columbus, OH, United States; ^4^ Department of Physiology and Cell Biology, College of Medicine, The Ohio State University, Columbus, OH, United States

**Keywords:** macrophage polarization, breast cancer, Slit2, immunometabolism, PyMT

## Abstract

Slit2 exerts antitumor effects in various cancers; however, the underlying mechanism, especially its role in regulating the immune, especially in the bone marrow niche, system is still unknown. Elucidating the behavior of macrophages in tumor progression can potentially improve immunotherapy. Using a spontaneous mammary tumor virus promoter-polyoma middle T antigen (PyMT) breast cancer mouse model, we observed that Slit2 increased the abundance of antitumor M1 macrophage in the bone marrow upon differentiation *in vitro*. Moreover, myeloablated PyMT mice injected with Slit2-treated bone marrow allografts showed a marked reduction in tumor growth, with enhanced recruitment of M1 macrophage in their tumor stroma. Mechanistic studies revealed that Slit2 significantly enhanced glycolysis and reduced fatty acid oxidation in bone marrow-derived macrophages (BMDMs). Slit2 treatment also altered mitochondrial respiration metabolites in macrophages isolated from healthy human blood that were treated with plasma from breast cancer patients. Overall, this study, for the first time, shows that Slit2 increases BMDM polarization toward antitumor phenotype by modulating immune-metabolism. Furthermore, this study provides evidence that soluble Slit2 could be developed as novel therapeutic strategy to enhance antitumor immune response.

## Introduction

Slit2, a secretory glycoprotein originally discovered for its role in neuronal guidance, is frequently reported to be deactivated by promoter methylation in several cancers, including breast cancer (1–4). Moreover, Slit2/ROBO1 signaling is reported to exert antitumor activity (5, 6). This antitumor effect is attributed to the regulation of β-catenin and chemotaxis (7). Recent studies also highlight the dual nature of Slit2 in cancer suppression or progression based on whether the tumor or surrounding cells produce and secrete this protein. Tavora et al. have recently shown that the deletion of Slit2 from the endothelium resulted in reduced extravasation of cancer cells and therefore metastasis, whereas Slit2 deletion in the tumor enhanced metastasis significantly (8). Another recent study describes the role of Slit2 in inhibiting macropinocytosis (9). Slit2 has also been shown to inhibit breast cancer by enhancing phagocytosis and reducing fibrosis (10). These studies suggest that Slit2 may play different roles in different cell types. However, the underlying mechanism by which Slit2 regulates immune cell metabolism, and its subsequent impact on breast tumor growth, has not been elucidated.

Immune cells in tumors play a crucial role in promoting tumor evasion, enhancing tumor growth, and even promoting metastasis by contributing growth factors, cytokines, and chemokines to create a pro-tumorigenic, immunosuppressive milieu called the tumor microenvironment (TME) (11). Slit2 is known to influence chemotaxis in eosinophils and neutrophils (12, 13). We have previously shown that Slit2 inhibits chemokine CXCL12-induced chemotaxis and chemoinvasion of breast cancer cells by inhibiting PI3K and MAPK activity (14).

Macrophages are innate immune cells known for their plastic nature and ability to respond to stimuli in order to polarize and elicit an appropriate immunological response. For instance, when encountered by a microbial infection, macrophages polarize to the classical M1 phenotype and rely on specialized functions, such as phagocytosis, to clear the infection. Similarly, when stimulated with pro-inflammatory signals in response to a wound, macrophages polarize to the alternative M2 phenotype and support the wound healing process (15). Pro-inflammatory M1 macrophages exert antitumor effects through phagocytosis and enhanced antigen presentation to engage T-cells (16). However, this ability is suppressed by cancer cells and even exploited to polarize macrophages to M2-tumor-associated macrophages (TAMs) that create an immuno-suppressive environment to support tumor progression and metastasis. M2 macrophages promote tumor progression by impairing the adaptive immune response, modulating cytokine production by macrophages, and stimulating angiogenesis and extracellular matrix remodeling (17). Besides differences in cell surface markers and function, there is a phenotypic distinction between M1 and M2 macrophages based on their cellular metabolism. Pro-inflammatory M1 macrophages primarily undergo glycolysis for their energetic needs, while enhancing fatty acid synthesis to generate anti-inflammatory eicosanoids. M2 macrophages, in contrast, rely on oxidative phosphorylation and fatty acid oxidation for their energetic needs (18–20).

While M1 macrophages are known to physiologically transition to M2 for wound healing, certain stimuli, such as antineoplastic agent paclitaxel, can reprogram M2 macrophages to M1 *via* TLR4 signaling (21, 22). While this capacity to reprogram pro-tumor M2 TAMs to antitumor M1 will eventually serve as a significant tool in cancer therapeutics, comprehensive *in vivo* studies exploring this paradigm are limited.

Recent research suggests that, even in the absence of metastasis to the bone marrow, breast tumor can reprogram myeloid cells in the bone marrow resulting in enhanced population of immunosuppressive neutrophils, and this has ramifications for tumor progression and aggressiveness (23, 24). However, little is known about the contribution of macrophages arising from the bone marrow niche in influencing antitumor or pro-tumor activity. Furthermore, while metabolites released from cancer cells are thought to stimulate and influence immune cells in the TME (15, 20, 25, 26), the potential impact of distal tumor cells on BMDM metabolism, and therefore polarization as M1 or M2 macrophages, is not fully appreciated. This is critical because monocytes from the bone marrow replace resident macrophages in most tissues over the course of the organism’s lifetime (27). Therefore, a strategy to modulate these cells and prime them for M1 polarization may act as a potent adjuvant to breast cancer treatment, particularly immunotherapy. This strategy may also overcome limitation of *in situ* reprogramming of TAMs, which is likely to be greatly affected by multiple systemic variables.

In this study, we show, for the first time, that Slit2 treatment enhances M1 polarization of BMDMs. Furthermore, we show a novel role of Slit2 in modulating antitumor immune response by altering BMDM metabolism. Considering the importance of TAMs in tumor development and metastasis (28, 29), targeting macrophages in the bone marrow represents a promising strategy to counter tumor progression, particularly in aggressive breast cancer subtypes.

## Methods

### Mouse Model and Treatment

Age-matched female transgenic tumor model mammary tumor virus promoter-polyoma middle T antigen (PyMT) (The Jackson Laboratory, USA) mice were used as murine model of breast cancer, and FVB (WT) (Charles River, USA) female mice as corresponding wild-type control. PyMT mice closely resemble aggressive human breast cancer development and have been thoroughly characterized for both pre-malignancy and malignant stages of breast cancer (30). While these transgenic mice show early molecular signs of hyperplasia (at 4 weeks) and carcinoma (at 10 weeks), palpable tumors are visible between 10 and 12 weeks of age. Beyond this age, the tumor develops aggressively and rapidly, thus making this an ideal time point to assess the efficacy of our treatment. All studies using mouse models were approved by the Institutional Animal Care and Use Committee at the Ohio State University (IACUC protocol number 2007A0233-R4).

At age 12 weeks, both PyMT and WT mice undergoing exogenous treatment with Slit2 received 5 μg/dose of recombinant Slit2 protein (R&D Systems, USA) dissolved in phosphate-buffered saline (PBS) intraperitoneally (i.p.), every alternative day for 2 weeks. This treatment strategy was based on a previous study reported by our group (10). The corresponding control group received an equal volume of PBS *via* i.p. on the same day. Tumor diameter was measured externally using a digital caliper, before and at the end of the 2-week treatment, and tumor volume was calculated as previously described (31). All mice were sacrificed at the end of treatment, and bone marrows were isolated for further experiments.

### Murine Bone Marrow-Derived Macrophage Isolation and Culture

The tibia and femur of mice were flushed under sterile conditions using PBS, and cellular materials were collected by centrifugation. These cells were then differentiated in media derived from L929 cells for 5 days to give rise to mature macrophages, as previously described (32).

To isolate myeloid cells for flow cytometry, the tibia and femur were flushed with PBS and resulting cells centrifuged. Additionally, for metabolomics analysis, these cells were incubated with anti-CD11b-labeled magnetic microbeads (Macs, Miltenyi Biotec, San Diego) and isolated using a magnetic stand as per supplier instructions.

Histological analysis in decalcified tibia and femur sections determined that these mice had no metastasis to the bone marrow (**Supplementary Figure 2**). Therefore, all assessments of metabolic parameters carried out in BMDMs are free of potential contamination from tumor/metastatic cells.

### Flow Cytometry

Single-cell suspensions of cells flushed from the bone marrow were used for flow cytometry analysis to identify the macrophage (CD45+ CD11b+ F4/80+), M1 macrophage (CD45+ CD11b+ F4/80+ CD38+), and M2 macrophage populations (CD45+ CD11b+ F4/80+ CDEGR-2+) using the LSRII instrument (BD Biosciences, San Jose, CA). The staining strategy to identify the immune-phenotype was modified from Cumming and Yu 2018 (33), and the antibodies used are listed in supplementary materials and methods (**Supplementary Tables 1–3**).

### Western Blotting

BMDMs that matured in culture were lysed in RIPA buffer and subjected to Western blot analysis, as previously described (34).

### Untargeted Metabolomics

Isolated CD11b+ cells were processed for metabolomics analyses using liquid chromatography-column isolation as described previously (28, 29). All sample preparation and QTOF analyses were performed by the Metabolomics Core Facility, Campus Chemical Instrument Center, at OSU. Metabolic cloud plots and PCA plots were generated using XCMS metabolomics tools (XCMS™, CA, USA). Human blood-derived monocytes were also analyzed, as described here. A brief description of the data processing strategy is provided in **Supplementary Materials and Methods 2**.

### Seahorse Bioanalyzer

BMDMs were subjected to Glycolysis Stress Test and Mito Stress test (as per Agilent Technology instruction) using the XF96 Extracellular Flux Analyzer (Agilent Technology, CA, USA). Data specifically describing change in ECAR after glucose injection were normalized to the steady non-glycolytic acidification rate induced by 2-deoxyglucose (2-DG, 50 mM). The baseline oxygen consumption rate (OCR) which represents mitochondrial activity has been normalized to residual OCR (ROX) in response to mitochondrial inhibitors antimycin and rotenone. The Glyco Stress test was also performed in PyMT BMDMs in the presence of D-galactose, a molecule that inhibits glycolysis, and an mTOR inhibitor (deforolimus).

### PyMT Whole-Body Irradiation and Allograft

To assess the impact of Slit2 on BMDMs, and subsequent tumor growth, one group of PyMT mice underwent total body irradiation at a sublethal dose of 8 Gray using a Gammacell 40 Exactor (137Cesium source, Best Theratronics), at a dose rate of 94 cGy/min. After 48 h, these mice were transplanted with bone marrow cells from a luciferase expressing Cre-reporter mouse (FVB.129S6(B6)-*Gt(ROSA)26Sor^tm1(Luc)Kael^*/J) (ROSA-Luc) purchased from Jackson Laboratory, USA. Once isolated from donor mice, these cells were treated *ex vivo* with PBS or recombinant Slit2 (100 ng/ml) for 24 h before injecting *via* the tail vein in irradiated myeloablated PyMT mice. This experiment allowed us to study the acute and direct effect of Slit2 on bone marrow monocytes independent of any systemic inflammatory or chemotaxis signaling. The optimization strategy of radiation dose is provided in **Supplementary Materials and Methods 3**. Recipient mice were sacrificed at age 14 weeks, and luciferase expression was tested in the bone marrow and tumor to assess homing for luciferase-expressing donor BMDMs to these specific sites.

### Human CD14+ Cell Isolation and Treatment

CD14+ cells were isolated from whole blood samples of five healthy control individuals purchased from a donor bank (Versiti Wisconsin Inc., Applied Research Laboratory, USA). Samples were acquired under approved no. IRB2019C0021. Leukocytes were isolated from whole blood samples as buffy coat and subjected to Classical Monocyte Isolation Kit (human) (Miltenyi Biotec) to collect label-free monocytes. These cells were then cultured and differentiated in 10% (v/v) plasma (from the same whole blood sample) in RPMI for 7 days, as described by Safi et al. (35). Upon differentiation, these cells were pretreated with recombinant human Slit2 (R&D Systems, USA) or PBS for 24 h, followed by a 24-h treatment with plasma samples from Triple-negative breast cancer patients (10% v/v) purchased from the Division of Human Genetics Sample Bank, Ohio State University. All samples were determined to have come from Caucasian women in the age range of 40–70 years, with no known metastasis, and were pooled for treatment purposes to reduce biological variability from individual patient plasma. At the end of the treatment, macrophages were washed and pelleted for metabolomics analysis.

### Statistical Analyses

All data are expressed as mean ± SEM, and statistical analyses were performed using ANOVA with Bonferroni *post-hoc* analysis, Student’s t-test, or paired t-test, as expressed in specific results. Differences were considered statistically significant at p<0.05. All statistical analyses were performed using the Prism software package version 4 (GraphPad Software, San Diego, CA, USA).

## Results

### Antitumor Effect of Slit2 and Enhanced Antitumor Macrophages in BMDMs

**Figure 1:**
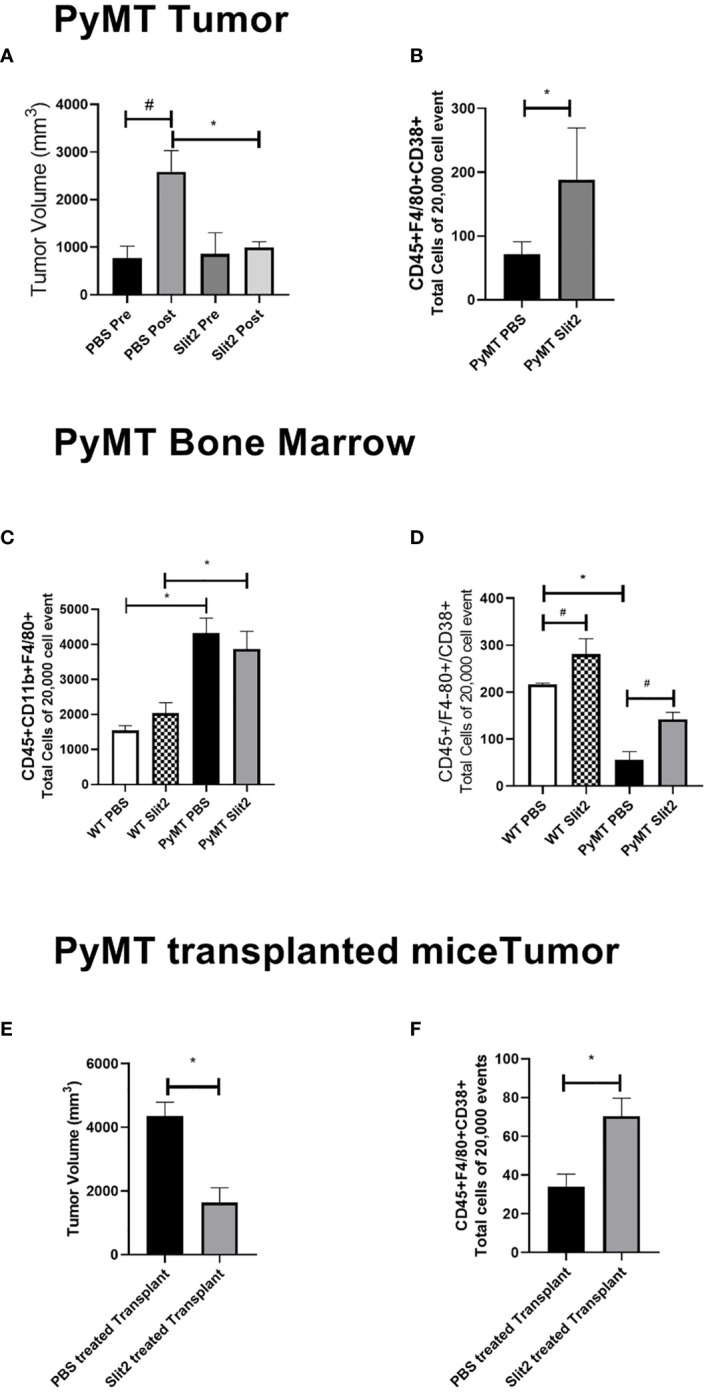
**(A)** Tumor volume measured before and at the end of PBS or Slit2 treatment in PyMT mice. **(B)** Tumor-associated population of CD45+ F4/80+CD38+ M1 macrophages in the arbitrary 20,000 events collected. **(C)** Bone marrow population of CD45+ CD11b+ F4/80+ macrophages in the arbitrary 20,000 events collected. **(D)** Bone marrow population of CD45+ F4/80+ CD38+ M1 macrophages in the arbitrary 20,000 events collected. **(E)** Tumor volume measured in myeoloablated PyMT transplanted with either Slit2-pretreated or PBS-pretreated allograft. **(F)** Tumor-associated population of CD45+ F4/80+CD38+ M1 macrophages in myeoloablated PyMT and transplanted mice. The images represent n = 3–5 mice/group. *represents p<0.05 significant difference between WT and PyMT mice treated with PBS or Slit2 as tested by unpaired t-test.

To assess the effect of Slit2 on tumor growth, we measured tumor volume in 12-week-old PyMT females (pre) and again after 2 weeks of i.p. Slit2 or PBS treatment (post). As shown in **Figure 1A**, PBS-treated PyMT (PBS pre mean ± SEM 776.04 ± 246.4 vs. PBS post 2584.8 ± 447.8 mm^3^, n = 5, #p < 0.005) mice showed an approximately 2.5x increase in tumor volume at the end of the study, whereas Slit2-treated mice did not show a significant change in tumor volume (Slit2 pre mean ± SEM 858.3 ± 442.1 vs. Slit2 post 997.4 ± 117.2 mm^3^, n = 5, p > 0.05), as tested by paired t-test. At the end of the treatment, PBS-treated PyMT mice had a significantly greater tumor volume than Slit2-treated mice as tested by unpaired t-test (*p < 0.005).

TAMs were analyzed using flow cytometry to determine the change in population of M1- and M2-polarized macrophages. While tumors from Slit2-treated mice only showed a trend in decrease in the M2 macrophage population (data not shown), M1 macrophages identified as CD45+F4/80+CD38+ cells were significantly more abundant in mice with Slit2-treated tumors compared to PBS-treated tumors (71 ± 20 vs. 188 ± 81.4 cells in 20,000 events, n = 4, *p < 0.05) (**Figure 1B**). This suggests that Slit2 attenuates tumor growth and progression, such that mice with palpable tumors do not show significant increase in tumor growth when treated with Slit2.

To assess the changes in the macrophage population in the bone marrow of PBS or Slit2 treated WT and PyMT mice, these cells were stained (CD45+CD11b+F4/80+) and quantified using flow cytometry. PBS-treated PyMT bone marrow appeared to have a larger population of total macrophages (4323.7 ± 424.3 cells/20,000 events) compared to PBS-treated wild-type mice (1540 ± 136.5 cells/20,000 events, n = 3, #p < 0.05) (**Figure 1C**). Slit2 treatment did not appear to affect total macrophage population in the bone marrows of PyMT (3869 ± 500.9) or WT (2036.3 ± 298.8 cells/20,000 events) mice. Interestingly, we observed that the M1 macrophage (CD45+F4/80+CD38+) population appears to be enriched in the bone marrow of WT mice compared to PyMT mice (216.3 ± 2.8 vs. 55.4 ± 18.2, n = 3–6, #p < 0.01) (**Figure 1D**). Furthermore, this population was enhanced in BMDMs of Slit2-treated WT (281 ± 32.8 cells/20,000 events) and PyMT mice (141.8 ± 15.3 cells/20,000 events), thus suggesting that Slit2 modulates the polarization of macrophages toward the antitumor M1 phenotype in the bone marrow. In contrast, the pro-tumor M2 macrophage population (CD45+CD11b+, F4/80+, ERG-2+) was marginally reduced in the bone marrow of PyMT mice compared to WT mice (not shown here); however, this difference was not statistically significant.

To assess the antitumor impact of Slit2 *via* changes in the bone marrow, independent of systemic effects, we studied the effect of Slit2 on macrophage polarization and overall tumor growth in PyMT mice that were irradiated with a sublethal dose to achieve myeloablation and later received an allograft with bone marrow cells from luciferase-expressing mice (ROSA-Luc). The bone marrow cells from these donor mice were treated with either PBS or mouse recombinant Slit2 in PBS. The sublethal dose of whole-body radiation that leads to over 90% CD45+ depletion in the PyMT bone marrow was determined at 8 Gray. Six-week-old PyMT females were subjected to irradiation at 8 Gray and subsequently received an intravenous transplant of bone marrow-derived cells harvested from ROSA-Luc mice treated either with PBS (PBS pretreated allograft) or with recombinant Slit2 (Slit2 pretreated allograft) for 24 h prior to transplant. This single dose of Slit2 resulted in significant impediment of tumor growth in PyMT mice. The tumor volume of PBS-pretreated allograft was 4,365 ± 424.2 vs. Slit2-pretreated allograft 1,643 ± 460, n = 6, *p < 0.05 (**Figure 1E**). Furthermore, we observed that while there was no change in luciferase-expressing cells homing in the bone marrow or tumor of PBS- and Slit2-pretreated allografted mice (**Supplementary Figure 1**), the Slit2-pretreated allograft-recipient PyMT had a higher population of F4/80+CD38+ M1 macrophages in tumors (75.7 ± 14.8% cells) compared to PBS-pretreated allograft-recipient PyMT tumors (22.1 ± 12.1% cells, n = 5, *p < 0.05) (**Figure 1F**). These results highlight the significant effect of Slit2 on BMDM polarization and its lasting effect on TME by enhancing M1 population in TAMs.

### Slit2 Enhances Flux Through Glycolysis in BMDMs

As discussed previously, immune cell metabolism greatly impacts their polarization and response, particularly to tumor cells. To assess whether Slit2 manipulates macrophage polarization by affecting glycolysis, we investigated the rate of glycolysis in BMDMs isolated from PBS- and Slit2-treated WT and PyMT mouse bone marrows using Seahorse Bioanalyzer. Briefly, Seahorse assay was used to assess ECAR at baseline (basal), and in response to glucose (glycolysis) and oligomycin (36). The flux in ECAR was normalized to non-glycolytic ECAR in response to 2-deoxyglucose. Basal ECAR was not significantly different in WT BMDMs compared to PyMT BMDMs (23.1 ± 3.8 vs. 15.9 ± 1.85 mpH/min, n = 5–15). BMDMs harvested from Slit2-treated mice showed enhanced ECAR in response to glucose (i.e., rate of glycolysis) in both WT (WT Slit2 37.5 ± 2.5 mpH/min, n = 5/group, #p < 0.05) and PyMT mice (PyMT Slit2 29.8 ± 3.1, n = 15/group, #p < 0.05), which is associated with the antitumor M1 macrophage polarization and is consistent with our flow cytometry outcome. Furthermore, ECAR in response to glucose, i.e., the rate of glycolysis in WT PBS BMDMs, was higher than PyMT PBS (42.7 ± 2.9 vs. 23.4 ± 2.08 mpH/min, n = 5–15, *p < 0.005). Slit2-treated WT BMDMs (51.4 ± 2.7 mpH/min) also showed significantly higher ECAR in response to glucose than both PBS (23.4 ± 2.08 mpH/min, n = 5–15, *p < 0.001) and Slit2-treated (32.6 ± 3.3 mpH/min, n = 5–15, *p < 0.005) PyMT BMDMs, as tested by one-way ANOVA. Slit2 treatment showed a significant increase in ECAR in both WT (**p < 0.05) and PyMT (**p < 0.05) BMDMs when tested by unpaired t-tested, assuming normal distribution. Glycolytic capacity, which is interpreted as the maximum capacity of cells to generate ATP in response to glucose solely by conversion to lactate or pyruvate, was elevated in WT compared to PyMT mice BMDMs and unaffected by Slit2 treatment.

**Figure 2:**
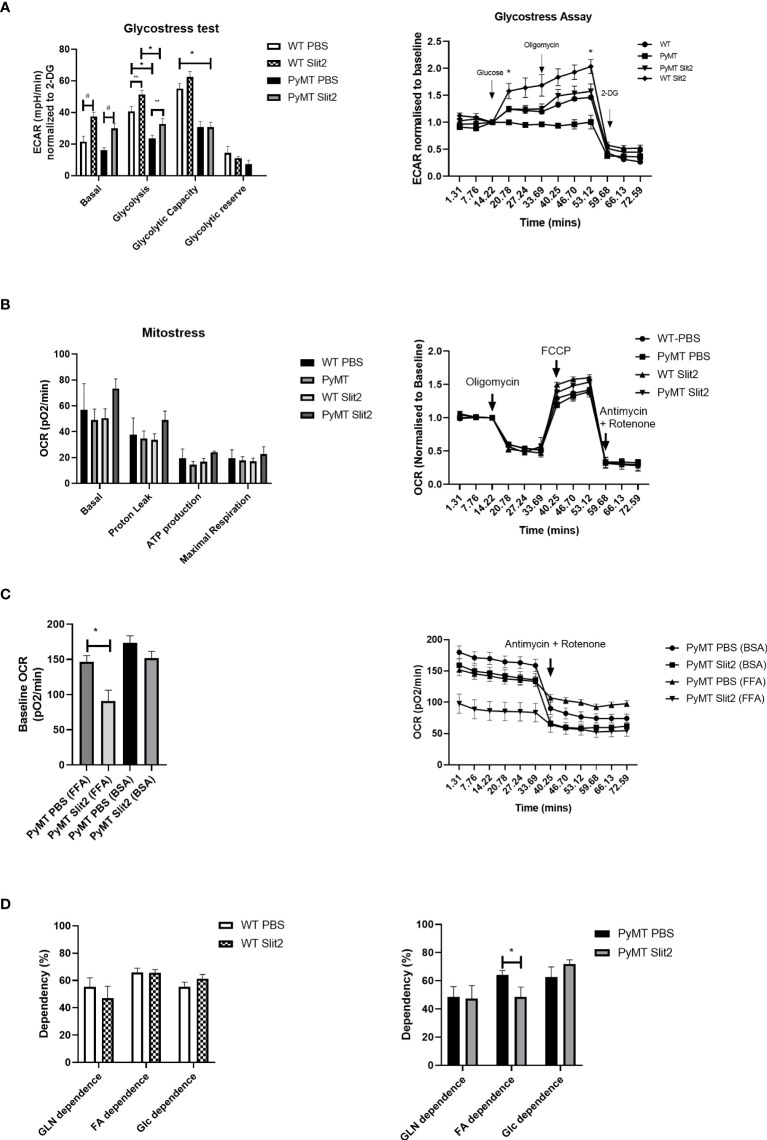
**(A)** Histogram of Glyco Stress test performed in BMDMs from PBS- and Slit2-treated WT and PyMT mice. The figure on the right represents kinetics of the assay, normalized to a steady reading at baseline. **(B)** Mito Stress test performed in BMDMs from PBS- and Slit2-treated WT and PyMT mice. The figure on the right represents kinetics of the assay, normalized to a steady reading at baseline. **(C)** Baseline oxygen consumption rate in response to pretreatment with BSA-conjugated free fatty acids. **(D)** Substrate dependence derived using the Mito Fuel Flex assay. The graph on the left represents the difference in substrate dependence between BMDMs from PBS- and Slit2-treated WT mice, while the graph on the right represents the difference in substrate dependence between BMDMs from PBS- and Slit2-treated PyMT mice. The images represent n=3–5/group. *p < 0.05, WT vs. PyMT, ^#^p < 0.05 PBS- vs. Slit2-treated groups based on one-way ANOVA, **p < 0.05 PBS- vs. Slit2-treated groups based on unpaired t-test.

Baseline OCR, which indicates mitochondrial activity, was unchanged between PBS- and Slit2-treated WT and PyMT BMDMs, as was flux in OCR in response to oligomycin and mitochondria membrane uncoupling agent, FCCP, between these groups (**Figure 2B**). However, *ex vivo* treatment of the PyMT BMDMs with BSA-conjugated free fatty acids (Agilent Technology) resulted in a significant increase in baseline OCR in PBS-treated PyMT mice. This response was attenuated in Slit2-treated PyMT mice (146.4 ± 8.7 vs. 90.9 ± 15.1 pO_2_/min, n = 12, *p < 0.05) (**Figure 2C**).

To further assess reliance on fatty acids, we performed the Mito Fuel Flex assay on PBS- and Slit2-treated mouse BMDMs. Here we found that WT PBS and Slit2 BMDMs showed no significant difference in dependence on glutamine (55.4 ± 6.4 vs. 47.0 ± 8.7%, n = 5), fatty acids (65.9 ± 3.07 vs. 65.6 ± 2.4%, n = 5), or glucose (55.3 ± 3.5 vs. 61.4 ± 3.09%, n = 5), while PyMT BMDMs showed a higher fatty acid dependence in PBS-treated mice compared to Slit2-treated mice (64.1 ± 3.1 vs. 48.5 ± 6.9%, n = 5, *p < 0.05). This suggests a possible reliance of PyMT BMDMs on mitochondrial fatty acid oxidation for cellular energetics, which is characteristic of M2 macrophages. Slit2 appears to reduce this effect while simultaneously enhancing glycolysis, which is the hallmark feature of antitumor M1 macrophages.

### Slit2-Mediated Glycolysis Is Dependent on mTOR Pathway

**Figure 3:**
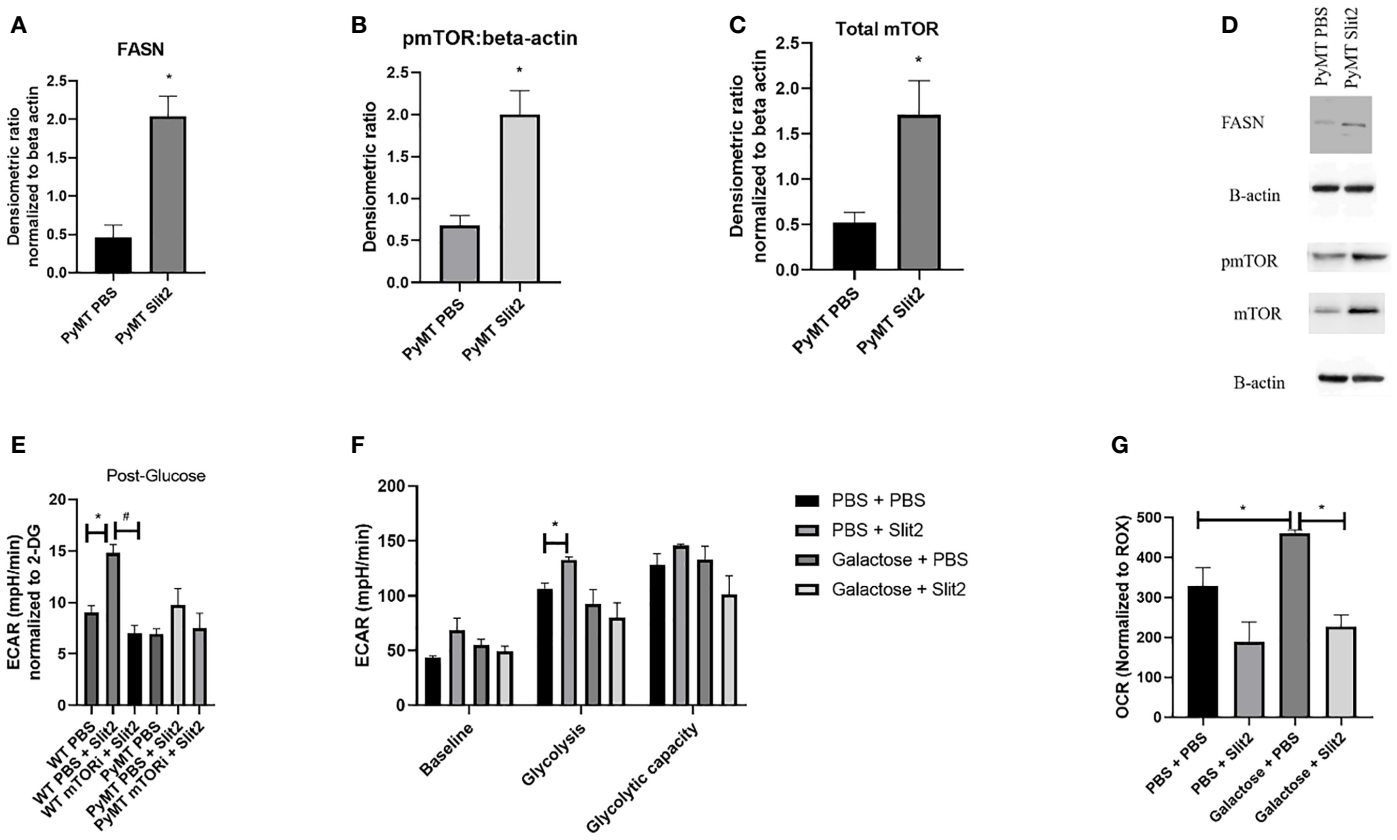
**(A)** Densitometric ratio of FASN. **(B)** Densitometric ratio of phosphorylated mTOR to beta-actin. **(C)** Densiometric ration of total mTOR to beta-actin. **(D)** Representative blots of Western blot analysis. **(E)** ECAR in response to glucose in BMDMs from WT and PyMT pretreated with mTOR inhibitor deforolimus (mTORi) and later treated with PBS or Slit2. **(F)** Glyco Stress test performed in BMDMs treated with Slit2 in the presence or absence of glycolysis inhibitor, galactose. **(G)** Baseline oxygen consumption rate in BMDMs treated with Slit2 in the presence or absence of glycolysis inhibitor, galactose. Images represent n = 3–6/group. *p < 0.05, assessed by unpaired t-test, ^#^p < 0.05 in mTORi and PBS pretreated cells.

To determine the potential mechanism by which Slit2 alters macrophage energetics and therefore polarization, we performed Western blot analysis in BMDMs isolated from PBS- and Slit2-treated PyMT mice, while we probed for key glycolysis regulating enzymes (hexokinase, phosphofructokinase, glyceraldehyde 3-phosphate dehydrogenase, and lactate dehydrogenase), we did not observe a clear statistically significant difference in the expression of these proteins. This is not entirely unexpected, as an increase in glycolysis can be a result of increased flux through the glycolytic machinery, without a major change in the protein expression of the involved enzymes. However Slit2-treated PyMT BMDMs showed elevated expression of fatty acid synthase (FASN) (0.46 ± 0.15 vs. 2.04 ± 0.26 densitometric ratio to beta actin, n = 5, p < 0.05) (**Figure 3A**). It has been reported that M1 macrophages synthesize fatty acids to use them as precursors to factors involved in inflammatory signaling such as eicosanoids (37, 38). The protein expression of pmTOR (Ser2448) (0.68 ± 0.1 PBS-treated vs. 2 ± 0.2 Slit2-treated, n = 3, p < 0.005) and total mTOR was significantly increased by Slit2 treatment (0.8 ± 0.07 vs. 1.08 ± 0.15, n = 3, p < 0.05) compared to PBS-treated PyMT BMDMs (**Figures 3B, C**). Representative blots of these proteins are presented in **Figure 3D**. mTOR is also known to regulate flux through glycolysis in immune cells (39).

To test the effect of mTOR on Slit2-mediated change in glycolysis in BMDMs, we pretreated both WT and PyMT BMDMs with PBS or mTOR inhibitor (deforolimus, MedChemExpress, USA). **Figure 3E** indicates that Slit2 treatment significantly increased ECAR in response to glucose (i.e., glycolysis) when compared to PBS-treated BMDMs (14.8 ± 0.7 vs. 9.03 ± 0.6 mpH/min, n = 6, *p < 0.05); however, this change was attenuated in BMDMs pretreated with mTOR inhibitor and later treated with Slit2 (7.03 ± 0.7 mpH/min, n = 6, #p < 0.05). While the same trend was seen in PyMT BMDMs, no statistical differences were observed. This may be due to the short-term treatment of BMDMs *ex vivo* with Slit2 compared to the longer *in vivo* treatment that altered glycolytic flux in BMDMs, as described in **Figure 2**.

Next, to appreciate the effect of Slit2 on macrophages through glycolysis, we performed the Glyco Stress test and assessed baseline OCR in BMDMs pretreated with galactose or PBS, followed by treatment with PBS or Slit2. Galactose is known to inhibit glycolysis and increase flux through mitochondrial oxidative phosphorylation in immune cells (40); here we hoped to see if galactose could reverse Slit2-mediated increase in glycolysis and decrease in basal OCR. While pretreatment with galactose did attenuate ECAR in Slit2-treated BMDMs, both at basal level (68.7 ± 10.9 vs. 55.2 ± 5.08 mpH/min, n = 3*p < 0.5) and in response to glucose (132.6 ± 2.9 vs. 92.4 ± 13.4 mpH/min) (**Figure 3F**), Slit2 treatment lowered baseline OCR in BMDMs pretreated with galactose (226.8 ± 29.3 vs. 461.3 ± 7.2 pO_2_/min, n = 3, *p < 0.05) (**Figure 3G**).

### Slit2 Mediates Changes in Metabolism in Human-Derived Monocytes Treated With TNBC Condition Media

**Figure 4:**
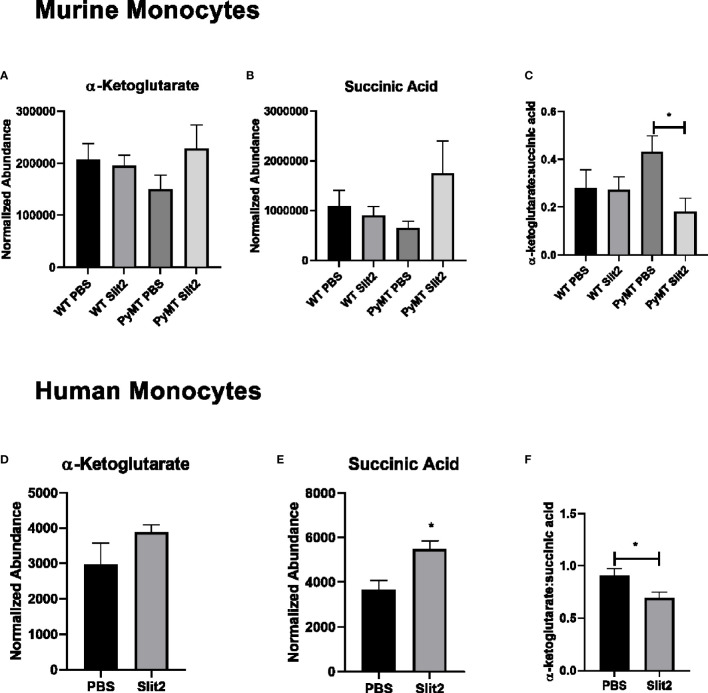
**(A)** Relative abundance of α-ketoglutarate in PBS- and Slit2-treated WT and PyMT mouse BMDMs. **(B)** Relative abundance of succinic acid in PBS- and Slit2-treated WT and PyMT mouse BMDMs. **(C)** Ratio of α-ketoglutarate:succinic acid in PBS- and Slit2-treated WT and PyMT mouse BMDMs. **(D)** Relative abundance of α-ketoglutarate in PBS- and Slit2-treated human BMDMs. **(E)** Relative abundance of succinic acid in PBS- and Slit2-treated human BMDMs. **(F)** Ratio of α-ketoglutarate: succinic acid in PBS- and Slit2-treated human BMDMs. Images represent n = 4–6/group. *p < 0.05, assessed by unpaired t-test.

To establish the effect of Slit2 on human monocytes and their metabolism, we first assessed untargeted metabolomics in murine CD11b+ cells. Based on PCA plots generated by XCMS, PBS- and Slit2-treated macrophages appear to have a distinct metabolic phenotype (**Supplementary Figure 3A**). When we compared key metabolites (citric acid, lactic acid, and glutamic acid) in monocytes derived from PBS- and Slit2-treated WT and PyMT mice, borderline changes were observed. However, Slit2 appeared to more distinctly change the abundance of α-ketoglutarate and succinic acid in murine bone marrow-derived monocytes (**Figures 4A, B**). It has been reported that a lower ratio of α-ketoglutarate to succinic acid enhances the pro-inflammatory M1 macrophage phenotype, whereas a higher ratio indicated higher M2 macrophage activity (15). We observed that the ratio of α-ketoglutarate to succinic acid was elevated in PyMT compared to WT mice (0.27 ± 0.07 vs. 0.43 ± 0.06, n = 3). Furthermore, this ratio is reduced in Slit2-treated PyMT mice (0.18 ± 0.05 *p < 0.05) (**Figure 4C**).

Next, we assessed the effect of Slit2 on cell metabolism in human macrophages, where monocytes isolated from whole blood (from age- and gender-matched, cancer-free volunteers) were matured in culture and pretreated with recombinant Slit2 or PBS, followed by treatment with TNBC patient plasma (10% v/v). We demonstrate here, for the first time, that Slit2 pretreatment of healthy human macrophages, followed by treatment with TNBC plasma, has a distinct metabolic phenotype from cells pretreated with PBS as represented by the PCA plots generated from untargeted metabolomic analysis of these cells (**Supplementary Figure 3B**). Furthermore, similar to murine macrophages, succinic acid was significantly more abundant in Slit2-treated human macrophages compared to PBS-treated cells (5,478 ± 374.4 vs. 3,679 ± 401.6 normalized abundance, n = 4, *p < 0.05) (**Figures 4D, E**). This also led to a significant decrease in α-ketoglutarate:succinic acid in Slit2-treated human macrophages exposed to TNBC condition media (0.91 ± 0.06 vs 0.69 ± 0.05, n = 4, *p < 0.05) (**Figure 4F**).

## Discussion

Slit2 is known to exert antitumor activity; however, the underlying mechanism, especially its role in priming of monocytes in the bone marrow, is not fully established. Recently, we have shown that recombinant Slit2 enhances the population of M1 macrophages in the tumor stroma, as well as phagocytosis, and subsequently tumor progression to metastasis (10). In our study, we observe that Slit2 influences the macrophage population in the bone marrow to enhance polarization toward the M1 macrophage population. Our data indicate that BMDMs from PyMT mice have a phenotype comparable to M2 macrophages, i.e., greater reliance on fatty acid oxidation and lower flux through glycolysis, which potentially contributes to and sustains an immunosuppressive TME, and aggressive progression of breast cancer in these mice. This is particularly interesting because in our model, macrophages in the bone marrow are influenced by Slit2. Therefore, Slit2-induced programming of bone marrow monocytes toward the M1 phenotype and their increased infiltration in tumor stroma potentially result in tumor attenuation.

Macrophages and their polarization are becoming an area of interest in immune therapy as evidenced by their key contribution to tumor progression. Macrophage infiltration of tumor serves as a prognostic marker of severity and outcome of breast cancer (41, 42). Furthermore, in murine models, macrophage depletion leads to favorable outcomes in tumor progression (43, 44). By transplanting myeloablated PyMT with Slit2- or PBS-treated allografts, we observed that Slit2 brought about changes directly in BMDM of tumor-bearing mice, which in turn enhanced their antitumor macrophage population and reduced tumor progression. While Casbon et al. (17) have provided evidence that neutrophils in the bone marrow of PyMT mice are involved in tumor progression, little is known about the communication between breast tumor and macrophage priming in the bone marrow. Nevertheless, this is an important phenomenon, since macrophages arising from the bone marrow home into the tumor and may sustain an immunosuppressive environment or, under treatment conditions, attack the tumor. Ongoing clinical trials such as NCT02183805 and NCT02670109 postulate that peripheral stem cell transplantation in combination with chemotherapy may be a superior therapeutic approach to treating heterogeneous and aggressive TNBC, a subtype of breast cancer with poor prognosis. Our novel observations suggest that Slit2-mediated antitumor macrophage modulation in the bone marrow may be a potential therapeutic option in treating aggressive tumors where a transplant may be indicated.

We also demonstrate, for the first time, that Slit2 influences BMDM cellular metabolism by promoting flux through glycolysis rather than mitochondrial oxidative phosphorylation. This is a first report showing that Slit2 may inhibit breast tumor growth and progression by modulating BMDM metabolic activity and thereby enhance antitumor macrophages in mice. Slit2 treatment enhances mTOR phosphorylation at Ser2448 as well as total mTOR expression in BMDMs. The effect of Slit2 on the mTOR pathway has never been elucidated in literature; however, mTOR is known to be highly sensitive to nutrient availability and AKT pathway and is a key regulator of metabolic reprogramming in several cells, including macrophages (45). Tuberous sclerosis complex (TSC)-deficient BMDMs that show higher mTOR expression and activity are reported to show higher M1 polarization (46–48). In addition, it has been reported that the AKT/mTOR/HIF1α axis is central to enhancing glycolysis and increasing “trained” response in macrophages by priming them for M1 polarization. The inhibition of mTOR or glycolysis can lead to diminished immune response to a *Candida albicans* infection (39). Furthermore, based on the Glyco Stress test performed in BMDMs treated with mTOR inhibitor (deforolimus), we have provided further evidence that the Slit2-mediated increase in glycolysis may depend on the mTOR pathway. Based on our findings, the Slit2-mediated increase in glycolysis *via* mTOR may prime or train BMDMs to polarize to the M1 phenotype more efficiently.

Furthermore, we have shown for the first time that Slit2 drives metabolic change in human macrophages in response to factors present in the plasma of TNBC patients, such that the abundance of succinic acid is enhanced while α-ketoglutarate:succinic is reduced by Slit2 treatment. The ratio of α-ketoglutarate to succinic acid has been reported to promote anti-inflammatory M1 macrophage polarization through epigenetic changes (19). This indicates a potential epigenetic effect brought about by Slit2 in sustaining glycolysis and therefore M1 macrophage phenotype.

Overall, this is a first report showing that Slit2 may inhibit breast tumor growth and progression by modulating BMDM metabolic activity and thereby enhance antitumor macrophages in TAMs of these mice. Furthermore, our studies indicate that Slit2 may represent a novel immuno-therapeutic approach against aggressive breast cancer by modulating the metabolism of macrophages.

## Data Availability Statement

The raw data supporting the conclusions of this article will be made available by the authors, without undue reservation.

## Ethics Statement

Ethical review and approval was not required for the study on human participants in accordance with the local legislation and institutional requirements. The animal study was reviewed and approved by the Institutional Animal Care and Use Committee at the Ohio State University.

## Author Contributions

KK conceptualized and designed the study, and performed experiments. KK also wrote the manuscript. MB provided technical expertise and support in data interpretation for the XFe Analyzer, and reviewed the manuscript. MY provided technical expertise and reviewed manuscript. DA provided support in maintaining the murine model and reviewed manuscript. SM provided support in maintaining the murine model and reviewed manuscript. KIS provided technical expertise and reviewed manuscript. NKJ provided technical expertise and reviewed manuscript. NCD provided access to the XFe Analyzer and critical review of manuscript. RKG conceptualized and designed the study, and wrote the manuscript. All authors contributed to the article and approved the submitted version.

## Funding

This work was supported in part by the National Cancer Institute, NIH, USA, grant no. R01CA109527 and DoD award W81XWH-19-1-0088 to RG; Pelotonia Postdoctoral award to KK, SM, and DA; and R01-HL138738 to KS. The authors would like to acknowledge the Comprehensive Cancer Center and Pelotonia for funding support.

## Conflict of Interest

NKJ serves as a consultant for Capture Collective Inc. RKG serves as consultant for Guidepoint consultation.

The remaining authors declare that the research was conducted in the absence of any commercial or financial relationships that could be construed as a potential conflict of interest.

## Publisher’s Note

All claims expressed in this article are solely those of the authors and do not necessarily represent those of their affiliated organizations, or those of the publisher, the editors and the reviewers. Any product that may be evaluated in this article, or claim that may be made by its manufacturer, is not guaranteed or endorsed by the publisher.

## Supplementary Material

Supplementary Figure 1 Luciferin expression/total cell in single cell suspensions prepared from bone marrow and tumor of PBS or Slit2 treated allografted PyMT mice.

Supplementary Figure 2 Representative images of H&E of Bone marrow to determine metastasis in WT and PyMT mice treated with PBS or Slit2.

Supplementary Figure 3
**(A)** PCA plot generated through untargeted metabolomics analysis of Slit2 and PBS treated PyMT BMDMs. **(B)** PCA plot generated through untargeted metabolomics analysis of Slit2 and PBS treated human macrophages exposed to TNBC condition media.
