# Supplementary figures and images for: Slit2-Mediated Metabolic Reprogramming in Bone Marrow-Derived Macrophages Enhances Antitumor Immunity
Source: Front Immunol. 2021 Oct 28;12:753477. doi: 10.3389/fimmu.2021.753477 (PMC8581492; doi:10.3389/fimmu.2021.753477)

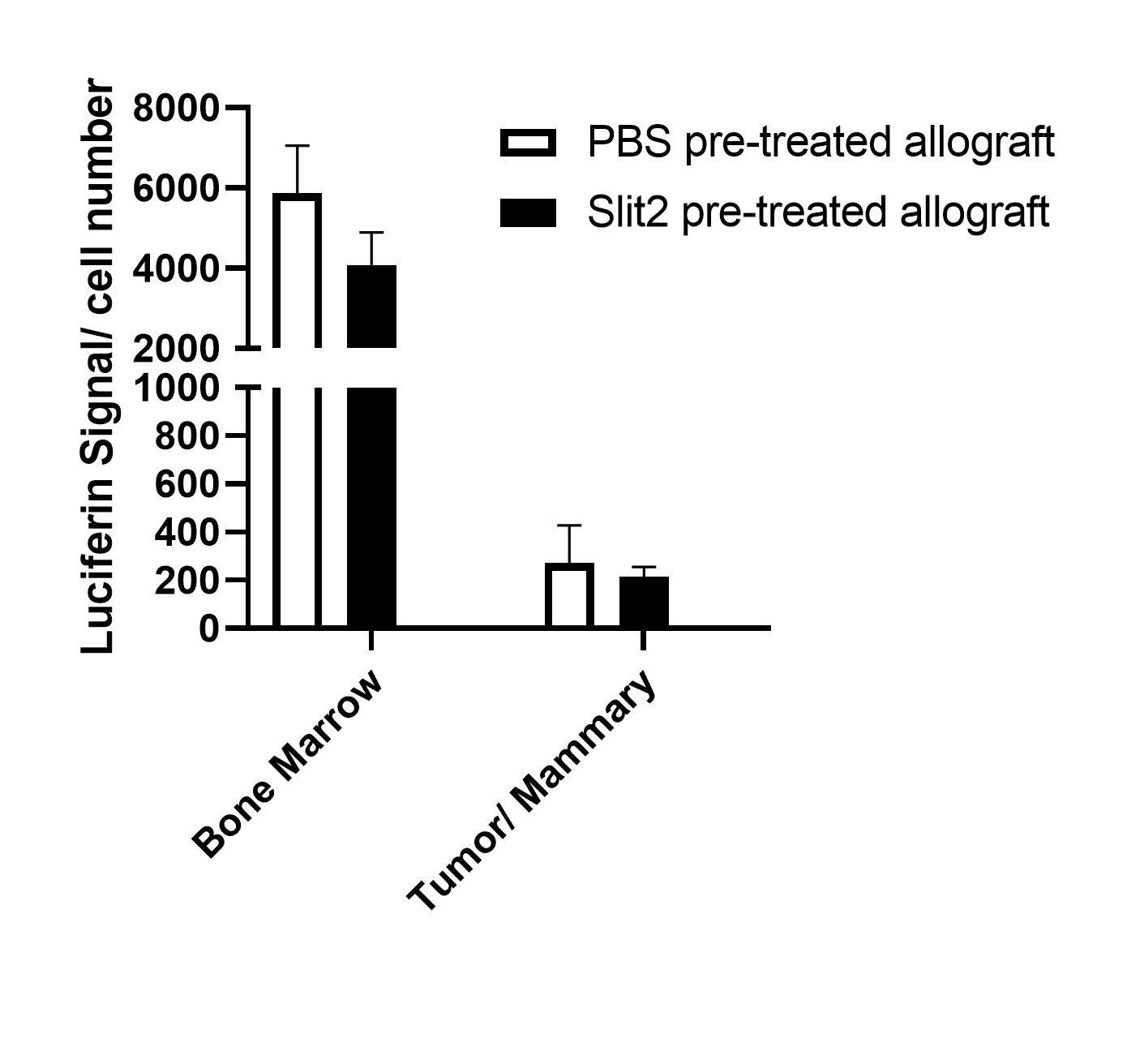

Supplement: Supplementary Figure 1 — Luciferin expression/total cell in single cell suspensions prepared from bone marrow and tumor of PBS or Slit2 treated allografted PyMT mice. [file Image_1.tif]

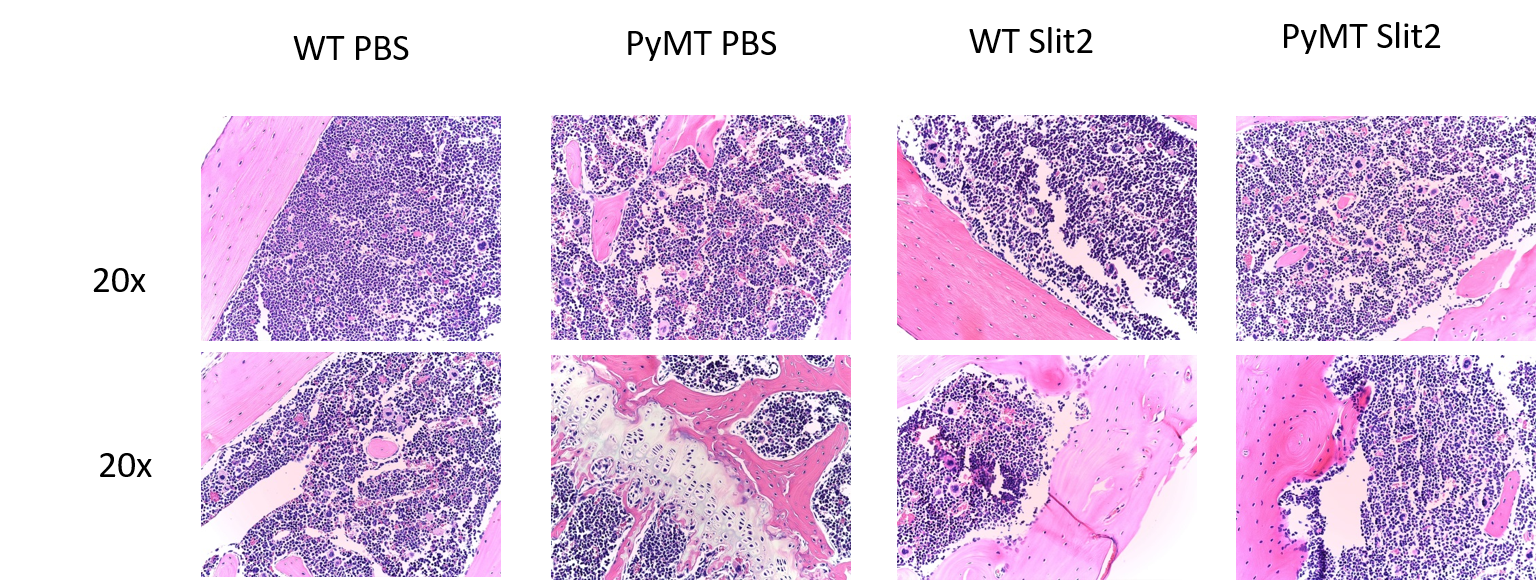

Supplement: Supplementary Figure 2 — Representative images of H&E of Bone marrow to determine metastasis in WT and PyMT mice treated with PBS or Slit2. [file Image_2.tif]

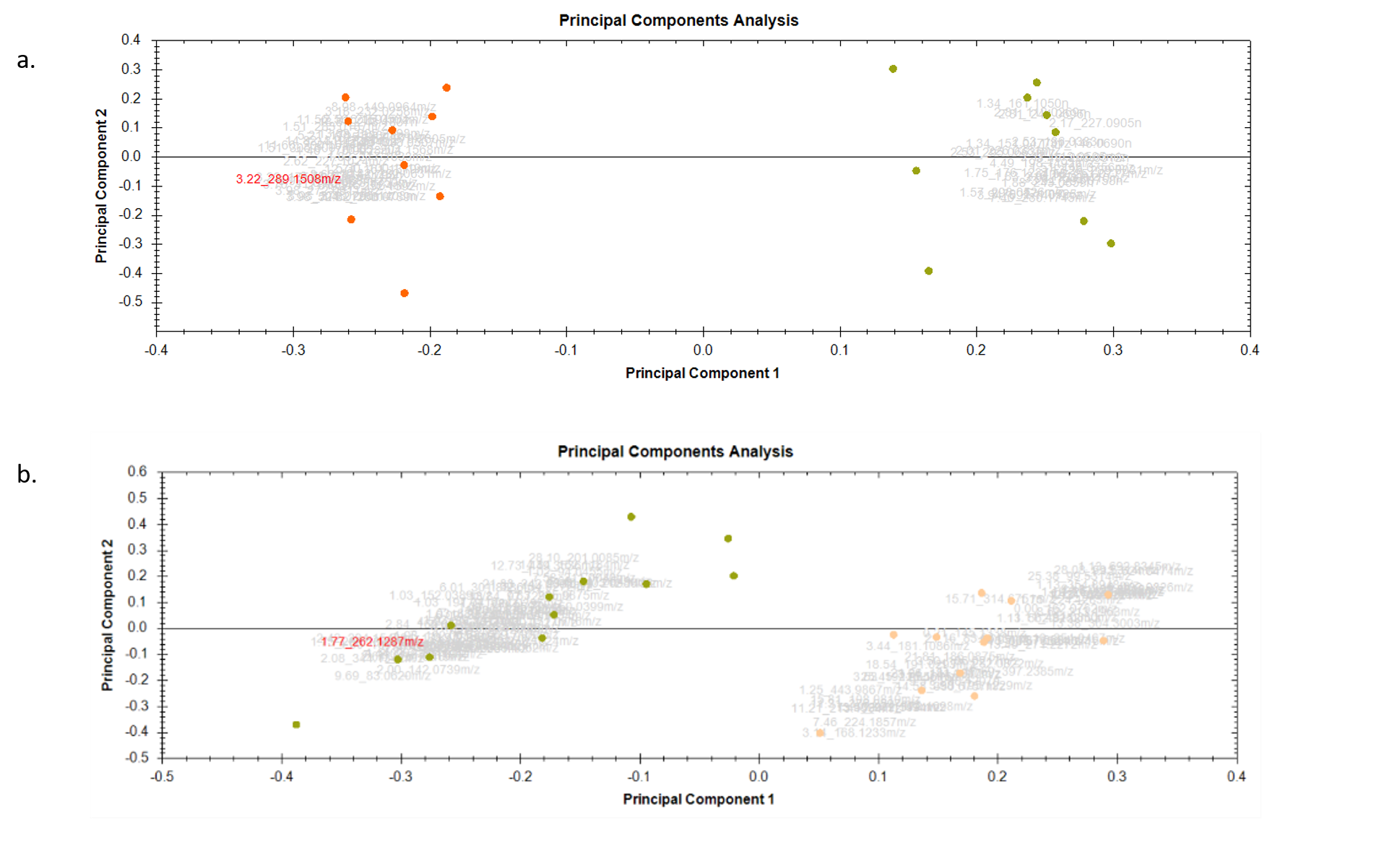

Supplement: Supplementary Figure 3 — (A) PCA plot generated through untargeted metabolomics analysis of Slit2 and PBS treated PyMT BMDMs. (B) PCA plot generated through untargeted metabolomics analysis of Slit2 and PBS treated human macrophages exposed to TNBC condition media. [file Image_3.tif]
