# Supplementary material for: Slit2-Mediated Metabolic Reprogramming in Bone Marrow-Derived Macrophages Enhances Antitumor Immunity
Source: Front Immunol. 2021 Oct 28;12:753477. doi: 10.3389/fimmu.2021.753477 (PMC8581492; doi:10.3389/fimmu.2021.753477)
Supplement: Supplementary file 4 [file DataSheet_1.docx]

**Supplementary Materials and Methods**

*FACS for immunophenotyping*

# Table 1 Panel1

| Target | Clone | Fluorophore | Supplier/ Cat. No. |
| --- | --- | --- | --- |
| CD45 | 30-F11 | Brilliant Violet 605 | Biolegend 103139 |
| CD11b | M1/70 | APC/Cy7 | Biolegend 101226 |
| F4/80 | BM8 | PE/Cy7 | Biolegend 123112 |
| IA/IE | M5/114.15.2 | Brillaint Violet 650 | Biolegend 107641 |

# Table 2 Panel 2

| Target | Clone | Fluorophore | Supplier/ Cat. No. |
| --- | --- | --- | --- |
| CD45 | 30-F11 | Brilliant Violet 605 | Biolegend 103139 |
| F4/80 | BM8 | PE/Cy7 | Biolegend 123112 |
| CD38 |  | FITC | ThermoFisher11-0381-82 |
| CD206 |  | APC | ThermoFisher 17-6691-82 |

**Table 3 Panel 3**

| Target | Clone | Fluorophore | Supplier/ Cat. no. |
| --- | --- | --- | --- |
| CD45 | 30-F11 | Brilliant Violet 605 | Biolegend 103139 |
| CD3 | 17A2 | APC | Biolegend 100236 |
| CD4 | GK1.5 | AF488 | Biolegend 100425 |
| CD8a | 53.67 | PE | Biolegend 100708 |

**Metabolomics data processing**

For feature selection in the untargeted results analysis, including database comparison and statistical processing, samples were analyzed in Progenesis QI and the pooled sample runs were selected for feature alignment with internal standard signals of heavy labeled ^13^C_3_-lactic acid (Avanti Polar Lipids) used to normalize between samples. All samples were aligned with a score of 90% or above and upon feature detection Anova p-value scores between the groups were calculated and a cutoff of 0.05. With database matching using the Human Metabolome Database, selecting for adducts M+H, M+Na, M+K, M+2H, for positive mode or M-H, M+Cl, and M-2H in negative mode and less than 10 ppm mass error, unique features were tentatively identified as potential metabolites. Unbiased PCA results were calculated from Progenesis.

**Optimization of whole body irradiation in PyMT mice**

FVB female mice of 6weeks were exposed to 9.0 Gray radiation, which led to significant toxicity by day 7. Therefore radiation dose was re-adjusted and tested at 8.0 and 7.0. Since 8.0 Gray was well tolerated, mice were sequentially sacrificed 24hours, 48hours, 72hours and 96hours post total body irradiation to determine gross evidence of radiation toxicity and myeloablation detecting CD45+ population in the bone marrow using flow cytometry. Since CD45 population was considerably diminished at 48hours, tail vein intravenous injections of transplant were planned for 48hours post irradiation for our experimental purposes.

*
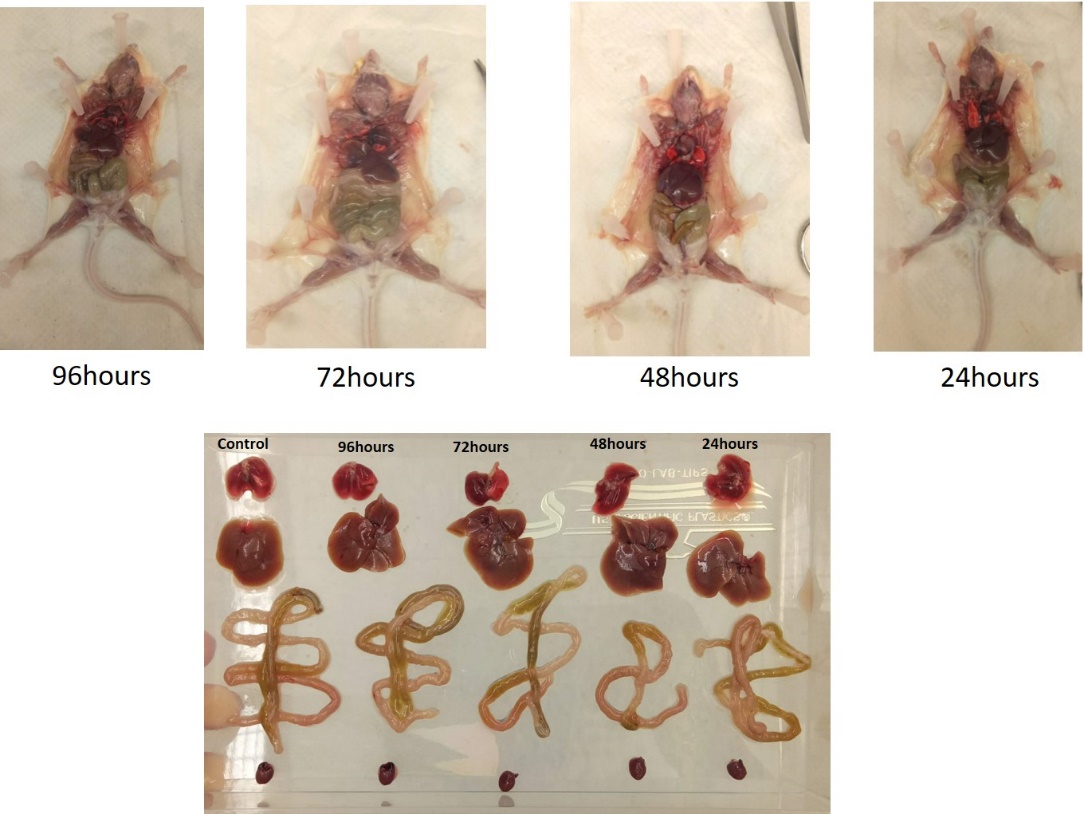
*

**Table 4.** Determination of CD45+ depletion in bone marrow

| *Name* | *Percentage Cells* |
| --- | --- |
| *Control* |  |
| *Total* | *90.2* |
| *Total/CD45+* | *27.4* |
| *24hours* |  |
| *Total* | *94.6* |
| *Total/CD45+* | *2.6* |
| *48hours* |  |
| *Total* | *90.2* |
| *Total/CD45+* | *0.74* |
| *72hours* |  |
| *Total* | *90.8* |
| *Total/CD45+* | *0.13* |
| *96hours* |  |
| *Total* | *90.6* |
| *Total/CD45+* | *0.056* |

*
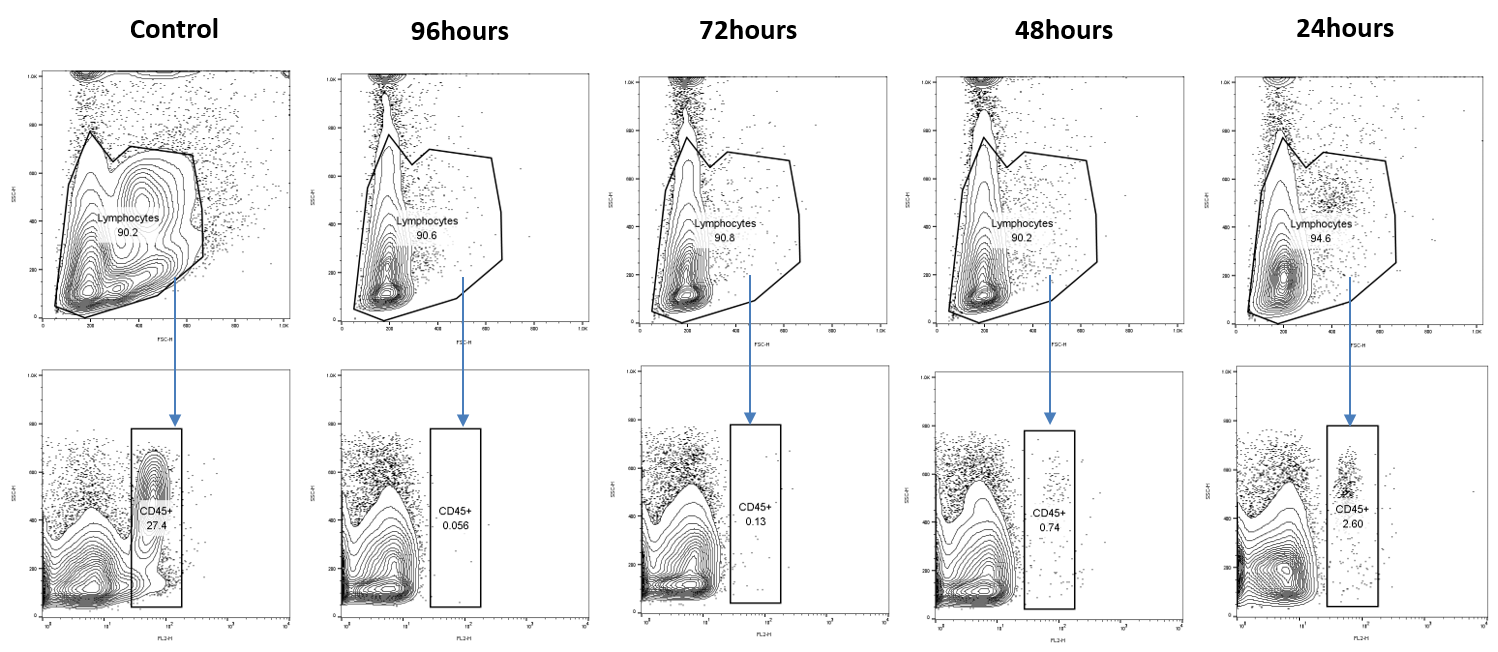
*

**Human CD14+ isolation and treatment**

Briefly, leukocytes were separated from whole blood samples as buffy coat by density gradient centrifugation using Histopaque-1077 (Sigma-Aldrich, USA). While the resulting plasma was isolated under sterile conditions, and used for culturing these cells, the buffy coat was subjected to Classical monocyte isolation kit (human) (Miltenyi Biotech) for negative selection of monocytes. The label free monocytes isolated through this process were cultured in 10% (v/v) plasma from the aforementioned healthy control blood in RPMI for 7 days as described by Safi et al [28]. Upon differentiation, these cells were serum starved and pre-treated with recombinant human Slit2 (R&D systems, USA) or PBS for 24 hours, followed by a 24 hour treatment with plasma samples from Triple negative breast cancer patients (10% v/v) purchased from Division of Human Genetics Sample Bank, Ohio State University. All samples were determined to have come from Causcasian women in the age range 40-70, with no known metastasis, and were pooled for treatment purposes to reduce biological variability arising from factors in the patient plasma. At the end of the treatment, cells were washed and pelleted for metabolomics analysis.
